# Crop rotation with *Meloidogyne*-resistant germplasm is useful to manage and revert the (a)virulent populations of *Mi1.2* gene and reduce yield losses

**DOI:** 10.3389/fpls.2023.1133095

**Published:** 2023-03-15

**Authors:** Aïda Magdalena Fullana, Alejandro Expósito, Nuria Escudero, Marina Cunquero, Pablo Loza-Alvarez, Ariadna Giné, F. Javier Sorribas

**Affiliations:** ^1^ Department of Agri-Food Engineering and Biotechnology (DEAB), Escola d'Enginyeria Agroalimentària i de Biosistemes de Barcelona (EEABB), Universitat Politècnica de Catalunya, BarcelonaTech (UPC), Castelldefels, Spain; ^2^ Institut de Ciències Fotòniques (ICFO), The Barcelona Institute of Science and Technology, Castelldefels, Barcelona, Spain

**Keywords:** *Citrullus*, *Cucumis*, pepper, plant resistance, root-knot nematodes, tomato

## Abstract

A rotation sequence of ungrafted and grafted tomato-melon-pepper-watermelon on resistant rootstocks ‘Brigeor’, *Cucumis metuliferus*, ‘Oscos’ and *Citrullus amarus*, respectively, was carried out in a plastic greenhouse, ending with a susceptible or resistant tomato crop. The rotation was conducted in plots infested by an avirulent (Avi) or a partially virulent (Vi) *Meloidogyne incognita* population to the *Mi1.2* gene. At the beginning of the study, the reproduction index (RI, relative reproduction in the resistant respect susceptible tomato) of Avi and Vi populations was 1.3% and 21.6%, respectively. Soil nematode density at transplanting (*Pi*) and at the end (*Pf*) of each crop, disease severity and crop yield were determined. Moreover, the putative virulence selection and fitness cost were determined at the end of each crop in pot tests. In addition, a histopathological study was carried out 15 days after nematode inoculation in pot test. The volume and number of nuclei per giant cell (GC) and the number of GC, their volume and the number of nuclei per feeding site in susceptible watermelon and pepper were compared with *C. amarus* and resistant pepper. At the beginning of the study, the *Pi* of Avi and Vi plots did not differ between susceptible and resistant germplasm. At the end of the rotation, the *Pf* of Avi was 1.2 the *Pi* in susceptible and 0.06 in resistant, the cumulative yield of grafted crops was 1.82 times higher than that of the ungrafted susceptible ones, and the RI in resistant tomato less than 10% irrespective of the rotation sequence. Concerning the Vi, *Pf* was below the detection level at the end of the rotation in resistant and 3 times *Pi* in the susceptible. The cumulative yield of grafted crops was 2.83 times higher than that of the ungrafted and the RI in resistant tomato was 7.6%, losing the population’s virulence. In the histopathological study, no differences in number of GC per feeding site were observed in watermelon compared to *C. amarus*, but they were more voluminous and contained higher number of nuclei per GC and per feeding site. Regarding pepper, Avi population did not penetrate resistant rootstock.

## 1 Introduction

Root-knot nematodes (RKN) *Meloidogyne* spp. are the most limiting plant-parasitic nematode genus for horticultural crop production worldwide (Hallman and Meressa, 2018). Four out of about 100 RKN described until now, *M. arenaria, M. incognita, M. javanica* and *M. hapla*, are the most damaging ones. These species are widely distributed around the world; they can parasitize a large number of plant species, and reproduce parthenogenetically (Subbotin et al., 2021). *Meloidogyne* spp. are sedentary endoparasitic nematodes of underground plant organs, mainly the roots. The nematode induces the formation of galls, which affect the proper absorption of water and nutrients by the roots and the plant productivity, mainly in intensive vegetable production systems under protected cultivation. Environmental and agronomical conditions favour the increase of nematode densities to be able to cause crop production losses (Sorribas et al., 2020). Maximum yield losses of vegetables due to root-knot nematodes under protected cultivation in the Mediterranean basin have been estimated at 62% in tomato, 86% in melon, 52% in pepper, and 37% in watermelon (López-Gómez et al., 2014; Giné and Sorribas, 2017a; Expósito et al., 2020; Talavera-Rubia et al., 2022).

Plant-parasitic nematodes control has been mainly based on chemical nematicides. Nonetheless, the number of active substances available has been progressively decreased due to their harmful effects on the environment and human and animal health (Sorribas et al., 2020). In addition, the use of nematicides has been limited to strictly necessary circumstances in the application of the European Directive 2009/128/EC, for the sustainable use of pesticides. Consequently, nematode management should combine durable and sustainable control methods, prioritising the natural regulatory elements to maintain nematode densities below the economic damage thresholds in an integrated pest management framework. In this context, plant resistance and agronomic practices are fundamental tools for nematode management. The genetic resistance is an effective and economically cost-effective technique against *M. arenaria*, *M. incognita* and *M. javanica* (Sorribas et al., 2005). Its use reduces the nematode population growth rate and the equilibrium density (Talavera et al., 2009; Giné and Sorribas, 2017a), resulting in a lower soil infestation at the end of the crop and significantly reducing the yield losses in the following crop in a rotation sequence (Ornat et al., 1997; Thies et al., 1998; Giné and Sorribas, 2017b). Genetic resistance for nematode control can be used through cultivars or rootstocks carrying resistance (R) gene(s). Unfortunately, resistant cultivars or rootstocks are only available for tomato (conferred by the *Mi1.2* gene), pepper (*N, Me1, Me3/Me7* genes), aubergine (*Solanum torvum*), and watermelon (*Citrullus amarus*) (Thies et al., 2010; Thies et al., 2015a; Thies et al., 2015b; Thies et al., 2016; García-Mendívil et al., 2019; García-Mendívil and Sorribas, 2021). Regarding melon and cucumber crops, despite some experimental rootstocks, such as *Cucumis metuliferus*, have been characterized as resistant to the most widely distributed RKN (Sigüenza et al., 2005; Kokalis-Burelle and Rosskopf, 2011; Guan et al., 2014; Gisbert et al., 2017; Ling et al., 2017; Expósito et al., 2019) there are not any available commercially at this time. Despite being an effective control method, the expression of some R genes can be affected by the genetic background of the plant and/or the RKN species or population (López-Pérez et al., 2006; Cortada et al., 2008), and the repeated cultivation of plant germplasm carrying the same R gene. It has been proved that the frequency of virulent individuals in a population increases progressively (Giné and Sorribas, 2017a) until the resistance is overcome after 2 or 3 consecutive crops with the same R gene, as it has been reported in tomato carrying the *Mi1.2* resistant gene (Verdejo-Lucas et al., 2009; Expósito et al., 2019) and pepper carrying the *Me3* resistance gene (Ros et al., 2006). However, the biological cost of acquiring virulence against specific R genes may lead to a decrease in the reproductive capacity of the nematode in susceptible genotypes of the same plant species (Djian-Caporalino et al., 2011). Therefore, the selection of virulence could be attenuated with rotation sequences including susceptible genotypes to achieve an acceptable production by the farmer, as proposed by Talavera et al. (2009), although monoculture contravenes the principle of sustainable production systems. Then, crop rotation sequences alternating different R genes could be a sustainable technique to improve resistance durability. In case of virulence selection to any specific R gene, the acquired virulence does not compromise other different R genes (Djian-Caporalino et al., 2011). Expósito et al. (2019) demonstrated that a 3-year rotation sequence with two different resistant sources (grafted tomato onto resistant tomato rootstock ‘Aligator’ and grafted melon onto *C. metuliferus*) was not enough to avoid virulence selection to a specific R gene, but the level of virulence was reduced. According to that, we hypothesized that including a greater diversity of R genes in a rotation sequence could reduce the risk of virulence selection or reverse it if it occurs. Therefore, the objective of the present study was to determine the effect of a 3-year rotation sequence including tomato, melon, pepper, and watermelon, ungrafted or grafted onto RKN-resistant germplasm on the *M. incognita* densities in soil and roots, the disease severity, the crop yield, and the putative selection for virulence in each resistant germplasm. In addition, histopathological studies were conducted to compare the volume and number of nuclei per giant cell (GC) and the number of GC, their volume and the number of nuclei at the feeding between susceptible watermelon and *C. amarus*, and between pepper and the resistant pepper rootstock.

## 2 Materials and methods

### 2.1 Crop rotation experiment

**Figure 1:**
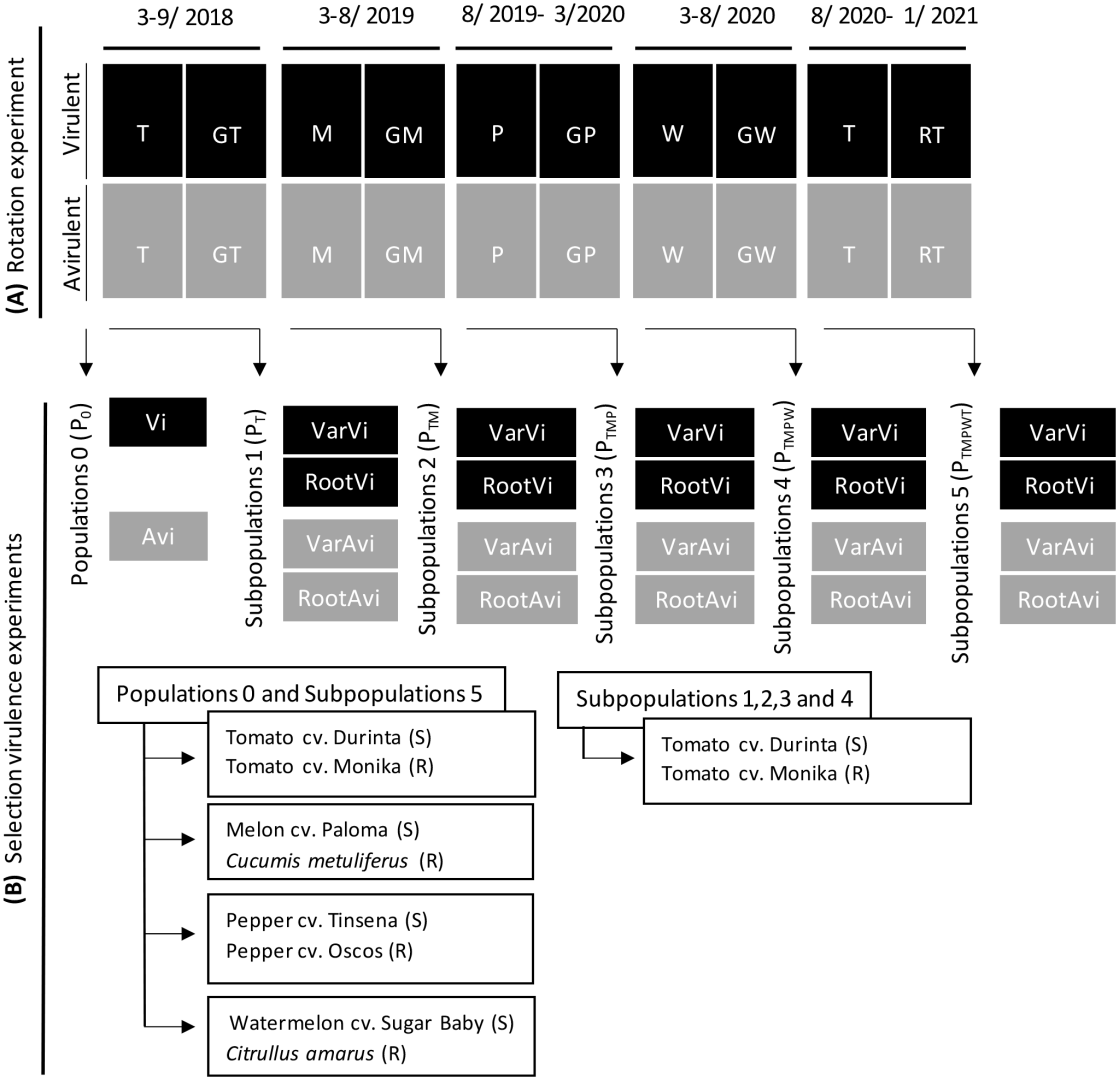
**(A)** Rotation schemes for ungrafted tomato (T) - melon (M) - pepper (P) - watermelon (W) or grafted onto the resistant rootstocks ‘Brigeor’(GT)- *Cucumis metuliferus* (GM) accession BGV11135- ‘Oscos’ (GP)- *Citrullus amarus* (GW) accession BGV5167 followed by a susceptible tomato cv. Durinta or resistant tomato cv. Caramba respectively in a plastic greenhouse infested with *Meloidogyne incognita* avirulent (Avi) or partial virulent (Vi) to the *Mi1.2* resistance gen. **(B)** Pot experiments conducted with the nematode populations (Avi and Vi) extracted just before the beginning of the rotation sequence (P_0_) and with avirulent (VarAvi and RootAvi) and partial virulent (VariVi and RootVi) subpopulations after each crop of the rotation scheme (P_T_-P_TM_-P_TMP_-P_TMPW_-P_TMPWT_) on susceptible and resistant cultivars or rootstocks.

The study was conducted in an experimental plastic greenhouse of 700 m^2^ in Viladecans (Barcelona, Spain) during three growing seasons (from 2018 to 2021). The soil texture was sandy loam with 83.8% sand, 6.7% silt, and 9.5% clay; pH 8.7; 1.8% organic matter (w/w); and 0.5 dS·m^-1^ electrical conductivity. The soil in the plastic greenhouse was solarised in the summer of 2014. After that, the soil was infested with a *Mi1.2* avirulent population of *M. incognita* and cultivated from 2015 to 2017 in two crop rotation sequences; tomato-melon or melon-tomato. The susceptible tomato cv. Durinta (Seminis Seeds, Missouri, USA), ungrafted or grafted onto the resistant tomato rootstock “Aligator” (Gautier Seeds, Eyragues, France) and the melon cv. Paloma (Fitó Seeds, Barcelona, Spain) ungrafted or grafted onto the resistant rootstock *C. metuliferus* accession BGV11135 (COMAV-UPV, Valencia, Spain) were used (Expósito et al., 2019). When the experiment finished, two nematode subpopulations were characterized for their level of virulence to the *Mi1.2* gene, one avirulent (Avi; reproduction index (RI; relative nematode reproduction in the resistant respect susceptible tomato) = 1.3%) and one partially virulent (Vi; RI = 21.6%) (Expósito et al., 2019). In this scenario, we conducted an experiment consisting of a crop rotation sequence of ungrafted and grafted tomato-melon-pepper-watermelon-tomato cultivated in both plots infested with an avirulent (Avi) and a partially virulent (Vi) nematode population (**Figure 1A**). The susceptibles tomato cv. Durinta, melon cv. Paloma, pepper cv. Tinsena (Enza Zaden Seeds, Enkhuizen, The Netherlands), watermelon cv. Sugar Baby (Batlle Seeds, Molins de Rei, Spain) ungrafted or grafted onto the resistant rootstocks ‘Brigeor’ (Gautier Seeds), *C. metuliferus* accession BGV11135, ‘Oscos’ (Ramiro Arnedo Seeds, Calahorra, Spain), and *C. amarus* accession BGV5167 (COMAV-UPV), respectively, were produced by the commercial nursery Hishtil GS (Malgrat de Mar, Spain). The resistant *Mi1.2* tomato cv. Caramba (De Ruiter Seeds, Bergschenhoek, The Netherlands) was used instead of the last grafted tomato crop of the rotation sequence due to the unavailability of the ‘Brigeor’ rootstock. Crops were cultivated in plots of 3.75 m^2^ (2.5 m length and 1.5 width) containing four plants spaced 0.55 m between them. Plots were spaced 0.9 m within a row and 1.5 m between rows. The experimental design in each of the areas infested by the avirulent or the partially virulent nematode population was randomized. The resistant and the susceptible germplasm were distributed randomly to the plots at the beginning of the experiment and they were maintained along the rotation sequence. Tomato was cultivated from March to September 2018, melon from March to August 2019, pepper from August 2019 to March 2020, watermelon from March to August 2020, and tomato from August 2020 to January 2021. Each combination of susceptible or resistant crop - (a)virulent population was replicated 10 times. The soil of each plot was carefully prepared to avoid cross-contamination. Plants were irrigated as needed *via* drip irrigation and fertilized with an NPK solution (15-5-30) at 31 kg·ha^-1^, iron chelate, and micronutrients at 0.9 kg ha^-1^. Fruits of each crop were harvested and weighed when they reached commercial standards according to the European Union commission regulation numbers 790/2000 (tomato fruits), 1615/2001 (melon fruits), 2147/2002 (pepper fruits) and 1862/2004 (watermelon fruits), and values were expressed as kg plant^-1^. At the end of rotation sequence, the cumulative yield of all grafted or ungrafted crops was calculated as the sum of the yield of each crop. Weeds were removed manually before and during the experiments. Initial nematode population densities were determined at transplanting (*Pi*) and at each crop’s end (*Pf*). Soil samples consisted of eight cores taken from the top 30 cm of soil with a 2.5 cm diameter auger. Then they were mixed and passed through a 4 mm-pore sieve to remove stones and roots. For each plot, *Meloidogyne* juveniles (J2) were extracted from 500 cm^3^ of soil using Baermann trays (Whitehead and Hemming, 1965) incubated at 25°C ± 2°C for 2 weeks. Afterwards, the J2 were collected using a 25 µm aperture screen sieve, counted, and expressed as J2 250 cm^-3^ of soil. At the end of each crop, roots were carefully removed from the ground, washed, and weighed, and then the galling index was evaluated on a scale from 0 to 10 –where 0 is a complete and healthy root system, and 10 is a dead plant (Zeck, 1971). After that, each plant root was cut into 0.5-1 cm pieces and homogenized, and two 20 g samples were used to determine the number of eggs. The eggs were extracted from roots by maceration in a 10% of commercial bleach solution (40 g L^-1^ NaOCl) for 10 min (Hussey and Barker, 1973), passed through a 74 µm-aperture sieve to remove root debris and collected on a 25 µm sieve. Eggs were counted in a Hawksley chamber under a compound microscope, and expressed as eggs plant^-1^. The remaining root samples of each nematode subpopulation-plant germplasm combination was mixed to obtain nematode inoculum to assess the selection for virulence in pot experiments (**Figure 1B**).

### 2.2 Selection for virulence experiments

Pot experiments were conducted in climatic chambers at the beginning of the plastic greenhouse experiment to determine the initial level of nematode (a)virulence to the resistant germplasm (experiment 1), and after each crop in the rotation sequence as indicator of putative changes along the rotation sequence (**Figure 1B**). The first experiment was conducted using J2 extracted from the soil at the beginning of the field experiment to corroborate the (a)virulence status of the nematode populations to the *Mi1.2* gene observed at the end of the 2015-2017 experiment previously described (Expósito et al., 2019). The resistant tomato cv. Monika (Syngenta, Basel, Switzerland), and the resistant rootstocks *C. metuliferus* BGV11135, *C. amarus* BGV5167 and pepper ‘Oscos’, and the susceptibles melon cv. Paloma, pepper cv. Tinsena and watermelon cv. Sugar Baby were used in the first and last experiments if enough nematode subpopulations inoculum was available. In the remaining experiments, only the resistant and susceptible tomato were used owing to the lack of nematode inoculum (**Figure 1B**). Seeds of *C. metuliferus* were germinated as reported in Expósito et al., 2018 and the rest of the plant seeds were sown in sterile vermiculite and maintained in a climatic chamber at 25 °C ± 2 °C and 16:8 h photoperiod (light: dark) for two weeks. Afterwards, plants were transplanted individually into pots (6.8 cm diameter and 8.2 cm high) filled with 200 cm^3^ sterile river sand and maintained under the same conditions. The nematode inoculum at the beginning of the crop sequence consisted of J2 extracted from the soil of plots with the avirulent (Avi) or partial virulent (Vi) population to the *Mi1.2* gene. The inoculum for the rest of the experiments consisted of J2 hatched from eggs produced on the resistant or the susceptible plant germplasm at the end of each crop of the rotation sequence. Thus, four subpopulations were obtained – VarAvi (from an ungrafted crop grown in plots infested with an avirulent population), VarVi (from an ungrafted crop grown in plots infested with partial virulent population), RootAvi (from a grafted crop grown in plots infested with avirulent population), and RootVi (from a grafted crop grown in plots infested with partial virulent population) (**Figure 1B**). Eggs were extracted from roots by maceration in a 5% commercial bleach solution (40 g L^-1^ NaOCl) for 10 min (Hussey and Baker, 1973), as previously described. The egg suspension was placed in Baermann trays at 25°C ± 2°C, and nematodes were collected daily for 7 days using a 25 µm sieve and stored at 9°C until inoculation. J2 obtained during the first 24 hours were discarded to ensure that J2 used as inoculum were not affected by bleach solution. Plants were inoculated with 200 J2 each when they had the third true leaf expanded. Plants were arranged randomly. Each plant species-subpopulation combination was replicated 15 times. Each experiment was conducted once at each time. Plants were watered as needed and fertilized with a slow-release fertilizer (15% N, 9% P2O5, 12% K2O, 2% MgO2, microelements; Osmocote^®^ Plus). Plants were kept in the climatic chamber for 40 days. Afterward, roots were carefully washed, and infectivity was assessed as the number of J2 able to infect and develop into egg-laying females; and expressed as the number of egg masses plant^-1^. The number of egg masses produced in each root system was counted after staining in 0.01% erioglaucine solution for 20 min (Omwega et al., 1988). Nematode eggs were extracted from the whole root of each plant and counted as described above, and expressed as the number of eggs plant^-1^. Fecundity was estimated as the number of eggs laid by each female and expressed as the number of eggs egg mass^-1^. The RI of each plant subpopulation was calculated as the percentage of the number of eggs per plant produced on resistant plants relative to that on susceptible plants for the same crop. The response was classified according to RI as highly resistant (RI < 1%), resistant (1% ≤ RI < 10%), moderately resistant (10% ≤ RI < 25%), slightly resistant (25% ≤ RI < 50%), or susceptible (RI ≥ 50%) (Hadisoeganda and Sasser, 1982).

### 2.3 Histopathology

**Figure 2:**
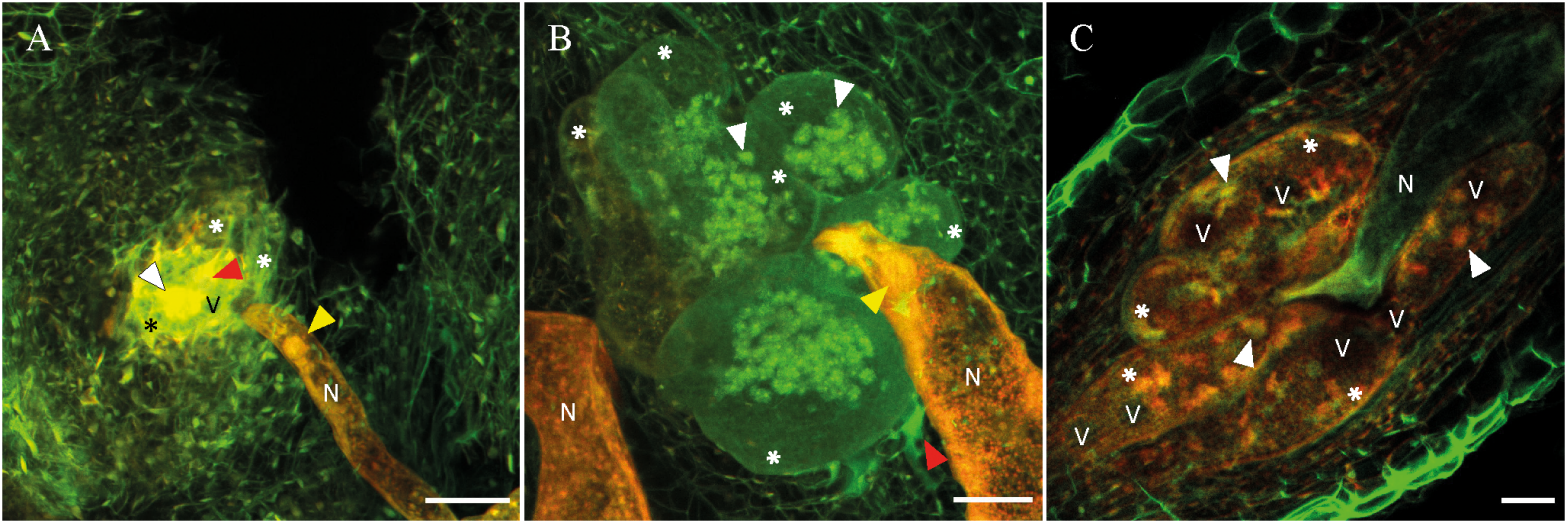
Laser scanning confocal microscope images of the infection site of *Meloidogyne incognita* 15 days after inoculation in the resistant *Citrullus amarus* BGV5167 **(A)**, and the susceptible cultivars watermelon cv. Sugar Baby **(B)** and pepper cv. Tinsena **(C)**. Nematode (N), vacuoles (V), giant cells (asterisk), some nuclei (white arrowhead), necrosed area (red arrowhead), and esophageal median bulb (yellow arrowhead) are indicated. Scale bar: 50 µm.

A histopathology study with laser-scanning confocal microscopy of cleared galled roots was carried out. Seeds of the susceptible watermelon cv. Sugar Baby and pepper cv. Tinsena and the resistant *C. amarus* BGV 5167 and pepper ‘Oscos’ were germinated under the same conditions described in the subsection 2.2. Once the second true leaf was expanded, 5 plants of each germplasm were transplanted into pots containing 200 cm^3^ of sterilized river sand. Seven days later, plants were inoculated with 200 J2 and 600 J2 of Avi *M. incognita* population in the susceptible or resistant germplasm, respectively. The nematode inoculum was obtained as previously described. The highest nematode density was used to inoculate the resistant germplasm in order to increase the probability to detect the nematode inside the roots. Conversely, the susceptible germplasms were inoculated with a low density to avoid coalescence of infection sites that could difficult the observation. The study was conducted once. Fifteen days after nematode inoculation, 10 root fragments per plant showing a single gall were selected. Samples were fixed, cleared, visualized and analyzed following the procedure described by Expósito et al. (2020) to determine the volume and number of nuclei per giant cell (GC) and the number of GC, their volume and the number of nuclei per feeding site. The visualized volume had a thickness ranging from 60 to 170 µm. Each volume was optically sectioned to produce a collection of Z-stack images (step size of 2-3 µm). For the GC volume measurement, images were manually segmented using the TrakEM2 ImageJ plugin (ImageJ, version 1.50i) that provides the giant cell area at each segment and calculates the volume of the structure. The volume of the feeding site was the sum of the volumes of all GC belonging to a feeding site. Representative frames of each plant germplasm are shown in **Figure 2** and **Supplementary Videos S1-S3**.

### 2.4 Statistical analysis

Statistical analyses were performed using the statistical software R V3.6.1 and the R Commander package (R Foundation for Statistical Computing, Vienna, Austria). Data from the field experiment concerning initial (*Pi*) and final (*Pf*) nematode densities in soil (number of J2 250 cm^-3^ soil), nematode reproduction (number of eggs plant^-1^) and disease severity (galling index) were compared between resistant and susceptible plant germplasms of the same crop per each Avi and Vi nematode population. Crop yield (kg plant^-1^) was compared between ungrafted and grafted plants per each crop and nematode population. Comparisons (*P* ≤ 0.05) were done either using the Student’s t-test if the data fitted a normal distribution or the nonparametric Wilcoxon test otherwise. The yield of the last tomato crop was not assessed due to the lack of information on the comparative performance and precocity between cultivars in the agri-environmental conditions in which the experiment was conducted.

Infectivity (number of egg masses plant^-1^), reproduction (number of eggs plant^-1^), and fecundity (number of eggs egg mass^-1^) data from pot experiments were compared between resistant and susceptible germplasm per each nematode population (first experiment) or subpopulations (experiments 2 to 6) to determine the putative selection for virulence. The nematode infectivity, reproduction, and fecundity were also pair compared between each nematode subpopulation with the VarAvi subpopulation (which was never exposed to resistant plant germplasm) to estimate the fitness cost to acquire virulence. The Student’s t-test (*P* ≤ 0.05) was used when data were normally distributed otherwise the nonparametric Wilcoxon test was used. The number of nuclei and GC per feeding site, the volume of each GC and the number of nuclei per GC were compared (*P* ≤ 0.05) between resistant and susceptible germplasm per each crop. Data were compared using the Student’s t-test if the data fitted a normal distribution or the nonparametric Wilcoxon test.

## 3 Results

### 3.1 Crop rotation experiment

The *Pi* of the avirulent or the partially virulent subpopulations at the beginning of the experiment did not differ between resistant and susceptible plant germplasms (**Table 1**). At the end of the rotation sequences, the *Pf* of the avirulent subpopulation was 1.2 greater than the *Pi* in plots cultivated with the susceptible germplasms but was 0.06 times the *Pi* where the resistant ones were cultivated. The reproduction of the avirulent subpopulation in the resistant germplasm ranged from 2.2% (tomato) to 39.6% (melon) of that achieved in the susceptible germplasm. Regarding disease severity, it only differed between the resistant and the susceptible tomato germplasm, being, in the former 0.2 and 0.26 times that of the registered in the susceptible one. In melon, 18.8% of the ungrafted plants died due to *Monosporascus cannonballus*, and the surviving ones showed a similar level of disease severity to that registered in grafted melon. In pepper, the root system developed poorly on both ungrafted and grafted plants and the galling index was not determined, but there were few nematodes that succeeded to reproduce. Grafted tomato and melon yielded 1.5 and 10.5 times more than the ungrafted ones but no differences between ungrafted and grafted pepper and watermelon yield were found. The cumulative yield of all grafted crops was 1.83 times more than that of the ungrafted at the end of the rotation sequence.

**Table 1:** Nematode soil densities at transplanting (*Pi*) and at the end of the crop (*Pf*), nematode reproduction (eggs plant^-1^), galling index and yield (kg plant^-1^) of the rotation sequence Tomato cv. Durinta (T)-melon cv. Paloma (M)-pepper cv. Tinsena (P)-watermelon cv. Sugar Baby (W), ungrafted or grafted onto the resistant rootstocks “Brigeor”(GT), *Cucumis metuliferus* (GM), “Oscos” (GP) and *Citrullus amarus* (GW), respectively, followed by a susceptible tomato cv. Durinta (T) or resistant tomato cv. Caramba (C) respectively, cultivated in a plastic greenhouse located at Viladecans (Spain) infested with a *Mi1.2* avirulent (Avi) and a partially virulent (Vi) *Meloidogyne incognita* populations from to 2021.

RKN population	Crop sequence	Cultivar/ Rootstock	*Pi* (J2 250 cm^-3^ soil)	*Pf* (J2 250 cm^-3^ soil)		Reproduction (Eggs (10^2^) plant^-1^)		GI		Yield (kg plant^-1^)	
**Avi**	Tomato (3-9/2018)	T	385 ± 116	3274 ± 1316*		6451 ± 1335*		7.1± 0.6*		3.1 ± 0.3*	
		GT	846 ± 200	532 ± 329		1021 ± 647		1.4 ± 0.3		4.6 ± 0.3	
	Melon (3-8/2019)	M	398 ± 104	332 ± 65		1744 ± 389*		4.3 ± 0.1		0.2 ± 0.01*	
		GM	243 ± 76	283 ± 76		690 ± 347		2.8 ± 0.6		2.1 ± 0.4	
	Pepper (8/2019- 3/2020)	P	332 ± 65	79 ± 46*		8 ± 2*		nd		0.2 ± 0.05	
		GP	283 ± 76	5 ± 2		0.2 ± 0.1		nd		0.4 ± 0.002	
	Watermelon (3-8/2020)	W	79 ± 46*	11 ± 6		20 ± 8*		2.3 ± 0.2		1.9 ± 0.5	
		GW	5 ± 2	12 ± 7		3 ± 3		1.6 ± 0.3		2.8 ± 0.5	
	Tomato* (8/2020- 1/2020)	T	11 ± 6	456 ± 124*		1024 ± 423*		3.8± 0.3*		nd	
		C	12 ± 7	48 ± 24		23 ± 11		1.0 ± 0.2		nd	
**Vi**	Tomato (3-9/2018)	T	154 ± 56	556 ± 285		4288 ± 1437		4.6 ± 1.2		4.2 ± 0.2*	
		GT	184 ± 74	332 ± 165		11164 ± 4651		3 ± 1		5.2 ± 0.2	
	Melon (3-8/2019)	M	166 ± 55	531 ± 67*		678 ± 289*		4.5 ± 0.5*		0.9 ± 0.3*	
		GM	87 ± 45	67 ± 24		101 ± 61		1.4 ± 0.5		3.3 ± 0.4	
	Pepper (8/2019- 3/2020)	P	531 ± 67*	63 ± 16*		19 ± 16		nd		0.3 ± 0.02*	
		GP	67 ± 24	1 ± 1		0.38 ± 0.08		nd		0.8 ± 0.001	
	Watermelon (3-8/2020)	W	63 ± 16*	8 ± 6		17 ± 10*		1.5 ± 0.4*		0.9 ± 0.4*	
		GW	1 ± 1	3 ± 3		0.03 ± 0.02		0.5 ± 0.2		8.5 ± 0.8	
	Tomato* (8/2020- 1/2021)	T	8 ± 6	469 ± 193*		357 ± 113*		3.3 ± 0.4*		nd	
		C	3 ± 3	0 ± 0		0.43 ± 0.29		0.1 ± 0.1		nd	

Data on nematode population densities in soil are the mean ± standard error of 10 replicates. Data on reproduction, Galls Index (GI), and yield are the mean ± standard error of 40 replicates. Values followed by * are different between grafted and ungrafted plants for each crop according to the Student-t Test or the non-parametrical Wilcoxon rank test (*P* < 0.05); GI: according to the scale of Zeck (1971), nd: Not determined; * Tomato cv. Caramba carrying the *Mi1.2* resistance gene was used as the last crop due to the commercial unavailability of the rootstock “Brigeor.”

Concerning the partial virulent population, the *Pf* at the end of the rotation sequence in plots cultivated with susceptible germplasms increased 3 times the *Pi*, whereas, in plots cultivated with resistant germplasm, the nematode was not detected in soil at the end of the rotation sequence (**Table 1**). The nematode reproduced 2.6 more times in grafted than ungrafted tomato (11164/4288). In the resistant tomato cv. Caramba the nematode reproduced 0.0012 times that achieved in the susceptible cv. Durinta (0.43/357). The disease severity was between 3 (watermelon) and 33 (last tomato crop) times higher (*P* < 0.05) in the susceptible than in the resistant germplasm, except in the first tomato crop, which did not differ. The grafted crops yielded between 1.24 and 9.44 more in the first tomato crop and the watermelon crop, respectively, than in the ungrafted susceptible genotypes. The cumulative yield of all grafted crops was 2.83 times more than that of the ungrafted at the end of the rotation sequence.

### 3.2 Selection for virulence experiments

**Figure 3:**
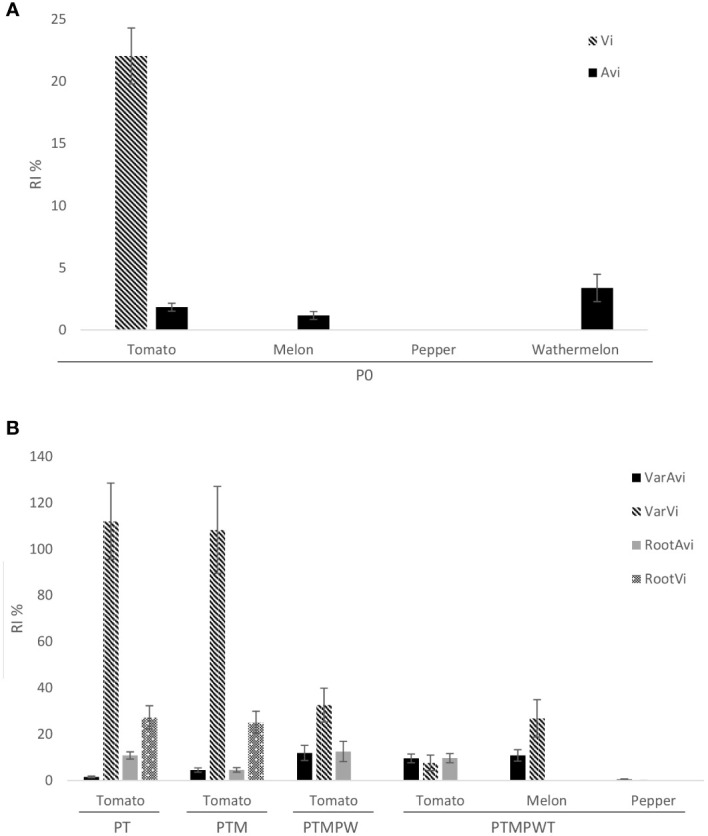
Reproduction Index (RI: percentage eggs plant ^-1^ produced in resistant germplasm respect those produced in the susceptible germplasm) **(A)** of *Mi1.2* avirulent (Avi) and virulent (Vi) *Meloidogyne incognita* populations obtained from soil before the rotation sequence (P_0_) in resistant tomato cv. Monika, melon rootstock *C. metuliferus*, pepper rootstock ‘Oscos’ and watermelon rootstock *C. amarus* and **(B)** of the *Mi1.2* avirulent (VarAvi and RootAvi) and partially virulent (VarVi and RootVi) subpopulation obtained from roots of each crop of the rotation scheme tomato (P_T_)- melon (P_TM_)- watermelon (P_TMPW_) in resistant tomato cv. Monika, and from roots of the last tomato crop (P_TMPWT_) in the melon rootstock *C. metuliferus* and pepper rootstock ‘Oscos’ too. The columns represent the mean and the bars represent the standard error of 15 replicates.

In the first pot experiment conducted with J2 from the soil just before starting the rotation sequence experiment, all the plant materials were assessed against the Avi population, but only resistant and susceptible tomato cultivars against the Vi population because of the lack of nematode inoculum. The RI of the Avi and Vi populations in tomato was 1.3% and 21.6%, respectively, confirming the avirulent and partially virulent status of the nematode populations (**Table 2**). Both Vi and Avi subpopulations showed lower (*P* < 0.05) infective (97.8% and 70.3%, respectively) and reproductive (98.7% and 78.4, respectively) capacity in the resistant than in the susceptible tomato cultivar. In addition, the fecundity of the Vi population in the resistant tomato cultivar decreased (*P* < 0.05) (**Table 2**). The RI of the Avi population in ‘Oscos’, *C. metuliferus* and *C. amarus* was 0%, 1.1% and 2.5%, respectively (**Figure 3A**). The infective and reproductive capacity, as well as the fecundity in the resistant germplasms were lower (*P* < 0.05) than in the susceptible ones (**Table 2**).

**Table 2:** N° of egg masses plant^-1^, egg plant^-1^ and eggs egg mass^-1^ produced in tomato cv. Durinta (S) and Monika (R), melon cv. Paloma (S) and *Cucumis metuliferus* (R), pepper cv. Tinsena (S) and ‘Oscos*’* (R) and watermelon cv. Sugar Baby (S) and *Citrullus amarus* (R) from the *Mi1.2* avirulent (Avi) and partially virulent (Vi) soil subpopulations of *Meloidogyne incognita* obtained before the rotation sequence in pot experiments.

Cultivar (host status)	Egg masses plant^-1^		Eggs (10^3^) plant^-1^		Eggs egg mass^-1^	
	Avi	Vi	Avi	Vi	Avi	Vi
Monika (R)	2 ± 0.27	27 ± 3.21	1 ± 0.24	16 ± 1.67	944 ± 169	628 ± 40
Durinta (S)	92 ± 2.98 *	91 ± 5.33 *	75 ± 3.36 *	74 ± 0.01 *	824 ± 30	831 ± 90 *
*C. metuliferus* (R)	2 ± 0.42	nd	0.7 ± 0.19	nd	322 ± 56	nd
Paloma (S)	50 ± 4.07 *	nd	60 ± 7.19 *	nd	1212 ± 115 *	nd
Oscos (R)	0 ± 0	nd	0 ± 0	nd	0 ± 0	nd
Tinsena (S)	34 ± 2.87 *	nd	25 ± 1.3 *	nd	754 ± 55 *	nd
*C. amarus* (R)	2 ± 0.42	nd	0.1 ± 0.004	nd	59 ± 15	nd
Sugar Baby (S)	6 ± 1.43 *	nd	4 ± 0.9 *	nd	568 ± 53 *	nd

Data is mean ± standard error of 15 replicates. Data followed by * are different between resistant and susceptible plants for each crop according to the Student-t Test or the non-parametrical Wilcoxon rank test (*P* < 0.05). nd, Not determined due to the low inoculum availability.

After the first tomato crop, four subpopulations of the nematode were differentiated, namely: VarAvi, RootAvi, VarVi and RootVi (**Figure 1B**). The VarAvi subpopulation remained avirulent to the resistance *Mi1.2* gene throughout the rotation sequence, and the RootAvi subpopulation finished the rotation with an RI of 10% after the last tomato crop (**Figure 3B**). The level of virulence of the VarVi subpopulation obtained at the end of the ungrafted tomato and melon crops was above 100%, decreasing progressively after the ungrafted watermelon (RI = 35%) and the last tomato crops (RI = 6.98%) (**Figure 3B**). The RootVi subpopulation maintained virulence levels of around 25% after the grafted tomato and melon crops (**Table 3**). At the end of the remaining crops in the crop sequence, not enough nematode inoculum was obtained for further evaluation. Concerning the fitness cost of acquiring virulence to the *Mi1.2* gene, less (*P* < 0.05) number of eggs per plant were produced by the VarVi subpopulation after tomato and melon crops, and the subpopulation RootAvi after the last tomato crop compared to those produced by the VarAvi subpopulation in susceptible tomato (**Table 3**).

**Table 3:** N° of egg masses plant^-1^, egg plant^-1^ and eggs egg mass^-1^ produced in the tomato cv. Durinta (S) and Monika (R) of *M. incognita* after each crop of the rotation scheme (tomato (P_T_)- melon (P_TM_)- watermelon (P_TMPW_)- tomato (P_TMPWT_)) on the cultivar and rootstock crop lines of the avirulent (VarAvi and RootAvi) and virulent (VariVi and RootVi) subpopulation, and those produced in the melon cv. Paloma (S) and *Cucumis metuliferus* (R) and in the pepper cv. Tinsena (S) and Oscos (R) from the root subpopulations obtained after the last tomato crop (P_TMPWT_) in 200cm^-3^ pot experiments.

Sub-population	Cultivar (host status)	Egg masses plant^-1^				Eggs (10^3^) plant^-1^				Eggs egg mass^-1^				
		VarAvi	VarVi	RootAvi	RootVi	VarAvi	VarVi	RootAvi	RootVi	VarAvi	VarVi	RootAvi	RootVi	
P_T_	Monika (R)	1 ± 0.2	11 ± 1.7†	19 ± 2.9†	16 ± 3.3†	0.8 ± 0.2	9 ± 1.3 †	12 ± 1.7 †	14 ± 2.6 †	608 ± 133	822 ± 76	678 ± 39	1010 ± 136
	Durinta (S)	49 ± 5.7*	34 ± 3.6*	134 ± 15.6*†	57 ± 5.7*	49 ± 4.1*	8 ± 0.7†	111 ± 9.9*†	51 ± 6.5*	1067 ± 80*	255 ± 27*†	924 ± 96	907 ± 76
P_TM_	Monika (R)	3 ± 0.4	8 ± 1.1†	3 ± 0.4	26 ± 3.5†	2 ± 0.5	12 ± 2.1†	2 ± 0.5	16 ± 3.1†	855 ± 117	1348 ± 1185	618 ± 96	606 ± 68
	Durinta (S)	59 ± 3.5*	28 ± 1.9*†	48 ± 4.3*	44 ± 4.9*†	49 ± 3.6*	11 ± 0.7†	48 ± 18.3*	64 ± 10.8*	848 ± 60	429 ± 28*†	1086 ± 126*	1444 ± 173*
P_TMPW_	Monika (R)	2 ± 0.3	2 ± 0.4	1 ± 0.1	nd	0.6 ± 0.2	0,7 ± 0.2	0.4 ± 0.1	nd	349 ± 75	270 ± 37	308 ± 84	nd
	Durinta (S)	10 ± 2.9*	10 ± 2.1*	5 ± 0.9*	nd	5 ± 2.0*	2 ± 0.6*	3 ± 0.4*	nd	422 ± 90	232 ± 41	541 ± 82	nd
P_TMPWT_	Monika (R)	5 ± 1.2	5 ± 1.7	20 ± 3.7†	nd	4 ± 0.7	3 ± 1.5	12 ± 2.4†	nd	906 ± 67	863 ± 280	596 ± 56,83†	nd
	Durinta (S)	138 ± 5.8*	65 ± 5.9*†	72 ± 6.6*†	nd	39 ± 5.3*	43 ± 3.9*	120 ± 5.9*†	nd	898 ± 36	656 ± 19†	529 ± 36†	nd
	*C. metuliferus* (R)	4 ± 0.6	5 ± 1.1	nd	nd	1 ± 0.25	1 ± 0.3	nd	nd	340 ± 79	276 ± 52	nd	nd
	Paloma (S)	24 ± 2.4*	16 ± 4.6*	nd	nd	10 ± 1.5*	4 ± 1.2*†	nd	nd	431 ± 38	576 ± 206	nd	nd
	‘Oscos’ (R)	0,4 ± 0.1	0.0001 ± 0	nd	nd	0.1 ± 0.0	0.004 ± 0.003	nd	nd	195 ± 49	36 ± 2†	nd	nd
	Tinsena (S)	17 ± 1.8*	39 ± 4.4*†	nd	nd	15 ± 1.1*	22 ± 2.5*†	nd	nd	952 ± 77*	574 ± 28*†	nd	nd

Data is mean ± standard error of 15 replicates. Data followed by * are different between resistant and susceptible plants for each crop according to the Student-t Test or the non-parametrical Wilcoxon rank test (*P* < 0.05). Data followed by † show signiﬁcant differences (*P* < 0.05) between VarVi, RootAvi and RootVi nematode subpopulations in compare to VarAvi subpopulation per plant according to the nonparametric Wilcoxon test (*P* < 0.05). nd, Not determined due to the low inoculum availability.

### 3.3 Histopathology

*Meloidogyne incognita* induced similar number of GC in *C. amarus* than in watermelon, but they were 8.9 times less (*P* < 0.05) voluminous in the former than in the later. The volume of GC per feeding site was 5.7 times higher (*P* < 0.05) in watermelon than in *C. amarus* (**Table 4**). In addition, GC in *C. amarus* had large empty vacuoles compared to watermelon (**Figure 2D**). The number of nuclei per GC and per feeding site were 7.7 and 20.2 times more (*P* < 0.05) in *C. amarus* than in watermelon (**Table 4**). Most of the induced GC in *C. amarus* presented few or no nuclei on it. Moreover, they were very difficult to image since the autofluorescence levels emitted were very low. The resulting images were dim in comparison with the resistant germplasms (**Figure 2**).

**Table 4:** Giant cell volume (GCV), GC volume per feeding site (GCV fs ^-1^), number of nuclei per GC (N GC ^1^), number of nuclei per feeding site (N fs ^1^), and number of cells per feeding site (NC fs _-1_) in the resistant plants (R) pepper rootstock ‘Oscos’ and *Citrullus amarus* and the susceptible plants (S) pepper cv. Tinsena and watermelon cv. Sugar Baby 15 days after nematode inoculation with 3 or 1 J2 cm ^3^ of soil, respectively, and cultivated in 200 cm^3^ pots in a growth chamber.

Cultivar (host status)	GCV (µm^3^ 10^5^)	GCV fs ^-1^ (µm^3^ 10^5^)	N GC ^-1^	N fs ^-1^	NC fs ^-1^
Oscos (R)	0 ± 0	0 ± 0	0 ± 0	0 ± 0	0 ± 0
Tinsena (S)	11.3 ± 1.1	81.9 ± 8.8	13.3 ± 1	96.5 ± 10.7	7.5 ± 1
*C. amarus* (R)	0.9 ± 0.2 *	9.3 ± 2.5 *	0.7 ± 0.6 *	6.5 ± 0.9 *	10 ± 1.1
Sugar Baby (S)	8.0 ± 1.1	54.2 ± 16.4	19.4 ± 2.8	131 ± 8.6	7 ± 0.8

Data are the mean ± standard error of 4 replications. Data in the same column followed by * indicates differences (*P* < 0.05) between *Citrullus* or pepper plants according to the non-parametric Wilcoxon test or Student’s t-test.

The nematode was able to infect and induce GC in the susceptible pepper cv. Tinsena, but no J2 were observed inside the root or GC in the resistant pepper rootstock ‘Oscos’ 15 days after nematode inoculation. Therefore, no comparisons between susceptible and resistant germplasm were carried out.

## 4 Discussion

The present work demonstrated that crop rotation, including at least four different sources of resistance to RKN, is efficient for managing both avirulent and virulent *M. incognita* populations to specific R genes, as well as, for reducing crop yield losses. In the current study, the *Mi1.2* gene in tomato, *Me3* gene in pepper and the resistant rootstocks *C. metuliferus* and *C. amarus* were included in the rotation sequence, assuming that each resistant plant germplasm has different plant defence mechanisms against the nematode and the risk to select cross-virulent populations is very low. Previous studies have shown that the level of resistance exhibited by resistant pepper carrying the *Me1* or *Me3* genes, *C. metuliferus* and *C. amarus* to virulent *Mi1.2* RKN isolates did not differ from that of avirulent ones (Castagnone-Sereno et al., 1996; Djian-Caporalino et al., 2011; Expósito et al., 2018; García-Mendívil et al., 2019). Therefore, different plant defence mechanisms can be induced by the nematode in those resistant plant germplasms avoiding the overlapping of signaling and the recognition of the resistance pathways that could result in cross-virulence selection (Petrillo et al., 2006). In tomato, the resistant *Mi1.2* gene induces localized cell death when J2 attempts to establish a feeding site (Williamson and Hussey, 1996) by preventing the production of enzymes that degrade or modify the cell wall and up-regulating the expression of genes encoding the defensin protein and protease, leading to phytoalexin production and proteolysis (Stotz et al., 2009). In addition, it induces the up-regulation of genes involved in the activation of signal transduction pathways, such as, receptor-like kinase and protein phosphatase. These actions result in the repression of giant cells formation, which are necessary to feed the nematode (Shukla et al., 2018). In pepper, the *Me3* resistant gene induces necrosis in cells of the root epidermis adjacent to the J2 by chlorogenic acid accumulation suppressing nematode penetration into the roots (Pegard et al., 2005). Regarding *C. metuliferus*, the reduction in J2 penetration and development has been associated with greater phenylalanine ammonia lyase and peroxidase activities along with the expression of several genes relevant for phenylpropanoid biosynthesis and plant hormone signalling compared to cucumber (Ye et al., 2017). Recently, 18 different root volatiles have been identified in *C. metuliferus* accession CM3 compared to cucumber, including hydrocarbons, alcohols, aldehydes, ketones and esters (Xie et al., 2022), which seems to be related to repelling J2 from roots. In *C. amarus*, the resistance has been associated with higher root fibrosity (Thies and Levi, 2003; Thies and Levi, 2007; Thies et al., 2016) and a different root metabolic profile, compared with watermelon, including amino acids, some of them reported to have nematicide effects, such as arginine (Sayed and Thomason, 1988); carbohydrates and several organic compounds (Kantor and Amnon, 2018).

Previous histopathological studies of the plant-nematode interaction conducted with laser confocal microscopy to compare between resistant and susceptible tomato cultivars, *C. metuliferus* and a susceptible melon cultivar or susceptible and resistant mutants of *Arabidopsis thaliana* revealed similar trends differentiating the resistant from the susceptible ones (Cabrera et al., 2015; Expósito et al., 2020). That is, the resistant germplasms had a greater number of GC per feeding site but smaller, less voluminous and with a lower number of nuclei, and some of the GC had no cytoplasm. In the current study, this trend was corroborated in the resistant *C. amarus* compared to watermelon, but comparisons were not possible in pepper owing to J2 infecting roots were not found in the resistant pepper rootstock ‘Oscos’ 15 days after nematode inoculation. In fact, a low percentage of plants were infected in the pot experiments conducted in the present study, and the nematode reproduced poorly in the plastic-greenhouse experiment. The defence mechanisms induced by the *Me3* gene previously described can explain these results.

Despite the effectiveness of plant resistance to manage RKN, after 3-years of monocropping resistant tomato or pepper, the level of resistance decreases or is null (Verdejo-Lucas et al., 2009; Ros-Ibáñez et al., 2014; Giné and Sorribas, 2017a). It is known that 2-4 years of rotation including non-host, poor-host and resistant-host is highly effective against *Meloidogyne* spp, but its effectiveness depends on the level of resistance of the plant germplasm (Trivedi and Barker, 1986), as well as the resistance source. Previous works have shown that 3-year rotation with two different sources of resistance, such as, tomato grafted onto ‘Aligator’ rootstock and melon grafted onto *C. metuliferus*, decreased yield losses caused by the nematode, but it did not prevent the selection for virulence to the *Mi1.2* resistance gene in tomato although it was attenuated (Expósito et al., 2019). The 3-year rotation sequence carried out in the present study with four different resistance sources has reduced the cumulative yield losses, has prevented the selection for virulence of an avirulent *Mi1.2* population and has reduced the nematode population density in the soil of a partially virulent population to undetectable levels. Interestingly, the level of virulence of the VarVi population decreased progressively after the melon crop from 100% to 7%. This subpopulation was exposed two times to resistant tomato germplasm during the period 2015-2017, but no fitness cost was detected, hypothesizing that a minimum of three resistant tomato crops would be needed to fix the trait (Expósito et al., 2019). Surprisingly, in the current study, the level of reproduction and fecundity of the females of VarVi in susceptible tomato was reduced compared to VarAvi -which was never exposed to resistant germplasm- when the inoculum produced in roots of the first tomato and melon crops were used, but not after the others. This event could be explained by a progressively decreasing proportion of virulent individuals in the population influenced by the following pepper and watermelon crops as well as the variability in infectivity, reproduction and female fecundity in the successive nematode generations. Petrillo and Roberts (2005) reported variability in the reproductive factors between isofemale lines, single descendent lines or isolates of virulent *M. incognita* to the *Rk* gene on susceptible cowpea even between nematode generations of the same origin. In fact, the subpopulation RootVi showed the same ability to reproduce on grafted and ungrafted tomato at the beginning of the plastic greenhouse experiment to reproduce poorly on resistant tomato cv. Caramba at the end of the rotation sequence, resulting in an insufficient nematode inoculum to be included in the virulence selection and fitness cost experiments.

Grafting improves crop yield (Gaion et al., 2018) and constitutes one of the most effective management methods to control soil-borne plant pathogens (Davis et al., 2008; Galatti et al., 2013), including RKN (Expósito et al., 2020). In our study, the cumulative yield of grafted crops at the end of the rotation sequence was higher than of ungrafted irrespective of the virulence status of the nematode population. Regarding watermelon and pepper crops, no differences in yield were found between grafted and ungrafted ones, possibly due to the poor host status and nematode tolerance of the former (López-Gómez et al., 2016) and the cropping season of the latter. In our conditions, pepper is transplanted from February to April instead of August as in the present study. The date of transplanting could influence the performance of the crop and the development of the nematode population, as has been reported by Vela et al. (2014) in zucchini squash.

The use of plant resistance is an effective and safe control method that has to be used properly in combination with other compatible and sustainable control methods to improve its durability. The level of the resistance expressed by a resistant plant germplasm depends on its background (Jacquet et al., 2005; Cortada et al., 2008). For instance, although all resistant tomato cultivars and rootstocks carry the *Mi1.2* gene, at least one additional locus is required for the expression of resistance (Martinez de Ilarduya et al., 2001). This fact could explain the differential response of some commercial tomato rootstocks and its influence in selecting virulent nematode populations (Verdejo-Lucas et al., 2009; Expósito et al., 2019). Understanding molecular plant-nematode interactions is needed to develop alternative approaches for nematode control (Abd-Elgawad, 2022). In addition to that, the use of plant resistance to a given nematode species could lead shifts in the plant-parasitic nematode communities. For example, cropping systems including resistant and susceptible crops and nematicidal cover crops designed for controlling RKN led to the replacement of RKN by Telotylenchidae nematodes (Mateille et al., 2020). Therefore, other control methods, such as, the use of cover crops, organic amendments, biological control agents, physical control methods or plant resistance inducers, such as *Trichoderma* species (Pocurull et al., 2020), *Bacillus firmus* (Ghahremani et al., 2020) or *Pochonia chlamydosporia* (Ghahremani et al., 2019) are necessary.

In summary, crop rotation with at least four different resistance sources is effective for the management of avirulent and partially virulent nematode populations to a given R gene and reduce crop yield losses.

## Data availability statement

The original contributions presented in the study are included in the article/**Supplementary Material**. Further inquiries can be directed to the corresponding author.

## Author contributions

FS, NE, AE and AG conceived, designed, supervised the experiments, the data collection, and analyses. AF performed the experiments, analysed the data, and wrote the draft of the manuscript. AF, MC and PL-A performed the histopathological study. AE, NE, MC, PL-A, AG and FS reviewed and wrote the final draft of the manuscript. All authors contributed to the article and approved the submitted version.
